# Experimental Durability Assessment and Multivariable Lifetime Modeling of UHMWPE Twisted String Actuators

**DOI:** 10.3390/ma19214695

**Published:** 2026-11-04

**Authors:** Jong-Hwan Yun, Yu-Jae Jeon, Min-Soo Kang

**Affiliations:** 1 Center for Contract-Based Degree Programs, Sun Moon University, 70 Sunmoon-ro 221beon-gil, Tangjeong-myeon, Asan-si 31460, Chungcheongnam-do, Republic of Korea; violet8087@sunmoon.ac.kr; 2 Interdisciplinary Studies, Yeoju Institute of Technology, 338 Sejong-ro, Yeoju-si 12652, Gyeonggi-do, Republic of Korea; superlittle@yit.ac.kr; 3 Department of Advanced Automotive Engineering, Sun Moon University, 70 Sunmoon-ro 221beon-gil, Tangjeong-myeon, Asan-si 31460, Chungcheongnam-do, Republic of Korea

**Keywords:** twisted string actuator, UHMWPE, cyclic durability, effective stress, stress-life scaling, frictional wear

## Abstract

Predicting cyclic durability while interpreting geometric and loading effects remains a challenge for ultra-high-molecular-weight polyethylene (UHMWPE) twisted string actuators (TSAs). This study analyzed 390 recorded lifetimes across 39 tested combinations of string diameter (1.0–2.0 mm), string number (two to seven), and applied mass (15–25 kg). A ten-coefficient second-order log-polynomial model was fitted to the logarithms of condition-wise arithmetic mean lifetimes and compared with a refitted power-law model. Lifetime increased with diameter and string number and decreased with applied mass. The second-order model achieved an in-sample mean absolute percentage error (MAPE) of 2.1212%. Leave-one-condition-out cross-validation yielded a MAPE of 2.8274%, compared with 3.4433% for the reduced model, a 17.89% relative reduction, although the maximum held-out error increased slightly. In the fitted surface, a positive diameter–mass interaction indicated greater proportional load sensitivity for thinner strings. Stress-equivalent local sensitivities were compatible with a nominal-stress interpretation at the reference condition, without establishing a universal stress–life law. Surface fuzzing, filament exposure, partial breakage, and pull-out were consistent with damage associated with repeated contact and relative sliding. The model provides a conditional engineering description within the tested domain; independent validation and confirmation of data assignments and record independence remain necessary.

## 1. Introduction

The rapid development of wearable robots, powered exoskeletons and prosthetic hands has created a growing demand for actuators that simultaneously offer high output, low weight and compact form factors [1,2]. Conventional electromagnetic motors and hydraulic systems provide excellent controllability and technological maturity, but their mass and volume often limit their integration into body-worn devices. Soft actuation technologies, including shape-memory alloys and pneumatic artificial muscles, offer attractive compliance, yet they are constrained by limited response bandwidth or the need for external pressure sources [3,4]. Against this background, twisted string actuators (TSAs) have emerged as a mechanically simple and highly effective actuation principle. In a TSA, a compact low-torque motor twists one or more parallel strings, and the resulting helical geometry converts rotational motion into axial contraction with a large mechanical advantage [5,6]. Their compact and lightweight characteristics make TSAs compatible with soft tendon-driven architectures and wearable robotic systems [7,8]. The performance of a TSA is governed fundamentally by the mechanical and tribological behaviour of the string material. 

Among the available candidates, ultra-high-molecular-weight polyethylene (UHMWPE) fibers, such as Dyneema^®^ SK75/SK78 (Avient Protective Materials B.V., Geleen, the Netherlands) and Spectra^®^ (Solstice Advanced Materials, Morris Plains, NJ, USA), are attractive for high-performance string and tendon applications owing to their high specific strength exceeding 3.0 N/tex, low fiber–fiber friction coefficient, excellent wear resistance, and chemical stability [9,10].

These properties allow UHMWPE-based TSAs to sustain repeated twisting–untwisting cycles under substantial tensile loads with relatively low energy dissipation. Owing to their high strength-to-weight ratio and flexibility, tendon- and cable-driven transmission elements have been widely considered for wearable robotic systems, including hand-wearable robots, lower-limb exoskeletons, and soft robotic suits [11,12,13]. Understanding and predicting the cyclic durability of UHMWPE-based TSAs is therefore essential for estimating the service life of the overall wearable system. Previous studies on TSA modelling can be broadly grouped into three categories. The first category concerns the kinematics and quasi-static mechanics of twisted bundles. Early studies established mathematical descriptions of twisted-string actuation and experimentally evaluated actuation behavior and force control [5,6]. Hanlan et al. [14] subsequently examined deviations from the standard twist–contraction model and showed that incorporating volume conservation improved agreement with experiments on nylon monofilament bundles up to intermediate twist angles. Their results highlight the importance of effective twist radius and bundle geometry in predicting axial contraction. These studies focus on actuation mechanics and contraction modeling rather than providing a cyclic lifetime model for UHMWPE-based TSAs. Zheng et al. [15] subsequently employed a backpropagation neural network to predict TSA displacement under variable loads for an anthropomorphic dexterous hand. This approach addresses load-dependent displacement prediction and motion generation rather than cyclic lifetime prediction. The second category focuses on load–torque coupling. Gaponov et al. [16] derived analytical relationships among motor torque, axial load and effective transmission ratio, showing that the required input torque is approximately proportional to both the applied mass and the effective radius of the twisted bundle. This line of work has helped establish load-dependent efficiency maps and has enabled the development of feedforward control strategies for TSAs. However, such models generally treat the string bundle as a time-invariant mechanical element and do not account for progressive degradation during repeated operation. The third category, which is most closely related to the present study, examines wear and lifetime behaviour under cyclic actuation. Usman et al. [17] experimentally investigated the cyclic lifetime of twisted string actuators and demonstrated that actuator durability is strongly affected by the applied mass and string configuration. Nguyen et al. [18] investigated TSA lifespan prediction using 144 valid samples, with string diameter, string number, and applied mass as the primary predictors. Their empirical power-law formulation used exponents of 2.509, 1.222, and −1.326 for diameter, string number, and applied mass, respectively, and had reported values of R^2^ ≈ 0.987 and RMSE ≈ 4962 cycles. Their hybrid approach combined the empirical formulation with XGBoost-based residual correction and achieved R^2^ = 0.9856, RMSE = 5299.47 cycles, and MAE = 3329.67 cycles, with a five-fold cross-validated R^2^ of 0.9752. The study also discussed feature importance, load–diameter coupling, and sensitivity, providing both predictive and interpretive insights. Because the present analysis uses condition-wise mean lifetimes and a different validation protocol, these published performance measures are not treated as directly comparable benchmarks for ranking the two studies. Beyond TSA-specific studies, recent investigations of synthetic-fiber and HMPE rope systems have further demonstrated that cyclic durability is governed by the combined effects of tensile loading, rope configuration, inter-fiber contact, and repeated relative motion. Lin et al. [19] experimentally investigated the dynamic-tension fatigue behavior of HMPE ropes and showed that fatigue life depended not only on the applied loading conditions but also on rope construction, highlighting the importance of internal structural stability and load distribution. Li et al. [20] subsequently examined HMPE ropes under cyclic bend-over-sheave loading and reported that lifetime decreased with increasing mean load, loading amplitude, and loading period, while repeated bending and contact produced coupled mechanical and thermal responses. More recently, Zeng et al. [21] emphasized the role of yarn-to-yarn contact and relative sliding in the wear behavior of synthetic-fiber ropes, showing that accumulated frictional interactions within the rope structure can contribute to progressive mechanical degradation. Xie et al. [22] further demonstrated that the mechanical response of full-scale HMPE ropes under cyclic loading depends strongly on mean load, load amplitude, and loading period, indicating that the behavior of braided polyethylene ropes cannot be represented solely by a single load parameter. Most recently, Lin et al. [23] directly investigated the sliding-wear mechanism of HMPE ropes and showed that tensile load and sliding conditions significantly affect surface wear, microfibril damage, and the degradation of mechanical properties. Collectively, these findings indicate that the durability of braided polyethylene structures is controlled by coupled stress, contact, friction, and geometric effects rather than by nominal tensile loading alone. However, how these mechanisms are quantitatively reflected in the lifetime scaling of UHMWPE-based twisted string actuators remains insufficiently understood. Against this background, three issues motivate further analysis of TSA lifetime data. First, building on existing multivariable models and interpretations of load–geometry interactions [17,18], the present study examines whether configuration-dependent lifetime sensitivities are statistically compatible with a common nominal tensile-stress description. This question is addressed through an explicit comparison of the variable-specific sensitivities with those implied by stress-only scaling, accounting for uncertainty and covariance in the fitted coefficients. The emphasis is therefore on evaluating a local scaling hypothesis rather than attributing physical mechanisms to coefficient signs or predictive accuracy alone. Second, post-failure morphologies can provide complementary qualitative context for interpreting lifetime trends, but their evidential role must be distinguished from quantitative validation of a lifetime model. Accordingly, the morphological analysis in this study is limited to documenting visible damage features and discussing plausible damage processes, without establishing a quantitative relationship between morphology, lifetime, and fitted coefficients. Third, the increased flexibility of a second-order lifetime model needs to be assessed against its predictive benefit under a common evaluation protocol. The present study therefore compares the reduced power-law and second-order models using leave-one-condition-out cross-validation of the same 39 condition-wise mean lifetimes. The objective is to assess internal predictive performance at the tested configurations, rather than to establish validated applicability limits or generalization to untested configurations and operating conditions.

Thus, the present study combines an explicit second-order log-polynomial lifetime model with covariance-based assessment of local sensitivities. First, the second-order formulation and a reduced power-law model are fitted to the same 39 condition-wise mean lifetimes and evaluated using identical leave-one-condition-out cross-validation, together with model-complexity criteria. Second, local logarithmic sensitivities are derived analytically and expressed as stress-equivalent quantities. Their equality is examined jointly at a specified reference configuration using the fitted coefficient covariance matrix. Conventional and heteroscedasticity-robust uncertainty estimates are also compared for the higher-order coefficients. Third, post-failure morphologies provide complementary qualitative context for the lifetime trends. Beyond adding quadratic terms, the analytical focus is on distinguishing predictive improvement from evidence concerning local nominal-stress compatibility and configuration-dependent response. Neither a supported interaction nor local agreement between sensitivities is interpreted as proof of a unique damage mechanism or a universal stress–life law.

## 2. Materials and Methods

Commercially available LIROS D-Pro braided strings (LIROS GmbH, Berg, Germany), manufactured from Dyneema^®^ SK78 UHMWPE fibers, were used in this study.

The manufacturer describes this product family as a coated, heat-set, 12-strand braid. Table 1 summarizes the nominal diameter, linear density, and manufacturer-listed breaking load for each of the three string sizes [24,25,26,27]. The linear densities were calculated from the manufacturer’s listed mass per unit length. These specifications describe the finished braided-string products and are distinguished from constituent-fiber properties. They are manufacturer reference values rather than measurements of the tested batch or of the knotted multi-string assemblies.

**Figure 1:**
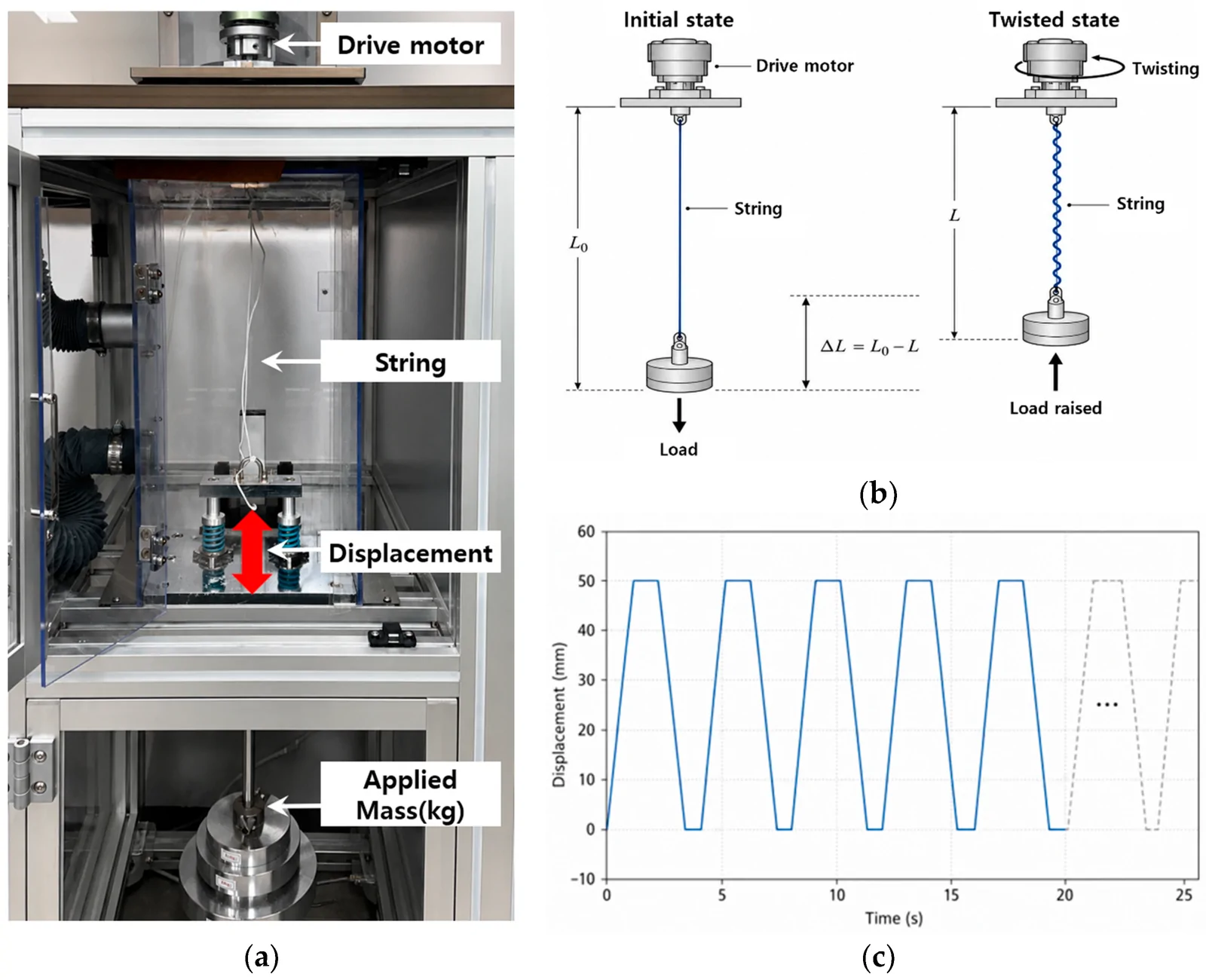
Experimental setup for the durability evaluation of the twisted string actuator: (**a**) test apparatus and loading configuration, (**b**) schematic illustration of the string contraction induced by twisting, and (**c**) displacement–time history applied during the cyclic durability test. In (**c**), the solid line represents the initial twist–untwist displacement cycles, while the dashed line indicates the schematic continuation of subsequent cycles. The ellipsis denotes omitted intermediate cycles.

Three commercial string sizes from the LIROS D-Pro SK78 product family, with nominal diameters of 1.0, 1.5, and 2.0 mm, were compared. The three sizes shared the specified Dyneema^®^ SK78 fiber grade and nominal 12-strand braid designation. Here, the 12-strand designation refers to the construction within each commercial string, whereas n denotes the number of separate strings assembled in the actuator. The common product designation does not establish identical constituent-yarn counts, yarn linear densities, or braid pitches across the three sizes. These diameter-specific construction parameters were not independently characterized for the tested specimens, and their equality across sizes is therefore not assumed. Accordingly, the experiment compares commercial string-size configurations rather than isolating geometric diameter as the only changing structural parameter. Each specimen consisted of the prescribed number of individual strings, with each string secured by separate overhand knots at both ends. A nominal free length of 250 mm was defined as the distance between the upper and lower string-fixing jigs, rather than the distance between the knots. A pneumatic lifting mechanism installed beneath the experimental apparatus was used to raise and position the loading assembly, allowing the jig-to-jig distance to be adjusted to 250 mm during specimen preparation. The knots were positioned at the terminal string-fixing regions to minimize their influence on the active twisting section. Heat-shrink tubing was applied to the knot regions to reduce local abrasion and prevent loosening. The cyclic test configuration is illustrated in Figure 1. A dedicated test procedure was used, with reference to general methodologies for fatigue testing of synthetic-fiber ropes [28]. One actuation cycle consisted of twisting through the prescribed condition-specific motor-rotation excursion and subsequently untwisting to the initial position. End of life was defined as a reduction of at least 20% in contraction displacement relative to the initial 50 mm reference under the prescribed condition-specific motor rotation, corresponding to a contraction displacement of 40 mm or less. The experimental system consisted of actuation, load application, and instrumentation subsystems. The actuation system employed a brushless DC servomotor with a rated speed of 3000 rpm and a rated torque of 1.27 N·m. A commercial servo drive with closed-loop control of position, speed, and current was used to operate the motor at 1500 rpm during twisting and untwisting. The motor shaft was connected to the upper end of the string through a precision universal coupling to minimize axial misalignment and unintended bending loads. Axial loading was applied by a static dead-weight system using masses of 15, 20, and 25 kg. These masses define the nominal loading conditions; the statistical model uses applied mass rather than a measured local filament stress. The axial contraction displacement, Δ*L*, was measured at 100 Hz using a linear variable differential transformer with a resolution of 1 μm mounted at the lower end of the string. A high-resolution optical encoder on the motor shaft was used to record the accumulated cycle count. Signals were recorded using an NI USB-6001 data acquisition module and an integrated C-based acquisition program.

The test matrix comprised 39 combinations, with ten recorded lifetime values per condition. The evaluated string-number ranges were 2–7 for *d* = 1.0 mm, 2–5 for *d* = 1.5 mm, and 2–4 for *d* = 2.0 mm. The upper limit on string number was reduced for the larger-diameter strings to avoid excessively large total nominal cross-sectional areas in the selected comparison set. Because the total nominal area increases with both string diameter and string number, the evaluated ranges were restricted to 2–7, 2–5, and 2–4 strings for nominal diameters of 1.0, 1.5, and 2.0 mm, respectively. This restriction was a practical choice of experimental configurations rather than an attempt to maintain equal total cross-sectional area across diameters. Consequently, the test matrix was not a complete factorial design, and the diameter and string-number predictors were correlated. The experimental sequence was organized into the following five steps. Step 1. Specimen preparation. The required number of strings was prepared and individually secured to the upper and lower fixing jigs. A pneumatic lifting mechanism was used to position the lower loading assembly and establish a nominal jig-to-jig distance of 250 mm. All strings were mounted using this common fixture spacing to maintain consistent initial geometric conditions. The end knots were located at the fixing regions, outside the main twisting section, and reinforced with heat-shrink tubing. Although this procedure provided a consistent assembly geometry, it did not independently verify equal tensile forces in the individual strings. Step 2. Pre-loading and zeroing. The prescribed tensile load, *F*, was statically applied for 300 s before cyclic actuation. The LVDT and optical encoder were then re-zeroed, and the baseline corresponding to the initial untwisted state was recorded. The 300 s hold provided a common pre-loading duration; it did not establish that creep had ceased or that time-dependent deformation would be negligible during the subsequent durability test. Step 3. Cyclic actuation. The motor rotation required to achieve an initial contraction displacement of 50 mm was determined separately for each experimental condition, depending on string diameter, string number, and applied mass. The prescribed rotation amount was then maintained throughout the repeated twist–untwist cycles for that condition, with a commanded motor speed of 1500 rpm. The contraction displacement was continuously monitored to detect performance degradation during cyclic operation. The prescribed rotation excursion was condition-dependent because the number of turns required to achieve the initial 50 mm contraction varied with string configuration and applied mass. The helix angle was not independently included as an experimental variable in the lifetime model. Furthermore, the commanded motor speed of 1500 rpm represents shaft rotational speed and should not be interpreted as the complete twist–untwist cycle frequency. The fitted lifetime relationships therefore apply to the prescribed actuation procedure rather than to independently controlled helix angles or cycle frequencies. Step 4. Failure detection and test termination. The end-of-life criterion was defined as a reduction of 20% or more in contraction displacement relative to the initial target of 50 mm under the prescribed condition-specific motor rotation. Accordingly, the test was automatically terminated when the contraction displacement decreased to 40 mm or less. The accumulated number of cycles at termination was recorded as the cyclic lifetime, *N_f_*. This displacement-based criterion was used to detect functional degradation associated with string damage or breakage, rather than requiring complete rupture of all strings. Step 5. Post-failure analysis. After the durability tests, the fracture regions and damaged string surfaces were examined using a VHX-7000 digital optical microscope (KEYENCE Corporation, Osaka, Japan) at a magnification of 20×. Representative specimens were selected for morphological observation to identify surface wear, filament damage, and fracture characteristics. The experimental conditions corresponding to the illustrated specimens are identified in the figure captions. The images were used for qualitative comparison of intact, cut, worn, and fractured regions; they were not used to reconstruct a time-resolved damage history. For statistical modeling, the arithmetic mean of the ten recorded lifetimes was calculated for each condition, and the natural logarithm of this mean was used as the response. The 39 condition means, rather than the 390 individual records, were the regression units. The second-order log-polynomial model included an intercept, three linear terms, three squared terms, and three pairwise interaction terms in normalized logarithmic diameter, string number, and applied mass. The reference values were 1.5 mm, four strings, and 20 kg. All ten coefficients were estimated jointly by ordinary least squares. A reduced four-coefficient power-law model, corresponding to the form previously used for TSA lifespan prediction [18], was refitted to the same response as a matched comparison baseline. Model definitions and numerical results are provided in Section 3.2. Both models were evaluated by leave-one-condition-out cross-validation: each condition mean was predicted from a fit to the other 38 conditions, and the procedure was repeated for all 39 conditions. No term selection or tuning was performed. Log-scale predictions were converted to cycles by direct exponentiation without a bias-correction factor. In-sample and held-out errors were evaluated separately, with log-scale fit statistics distinguished from cycle-scale prediction statistics. Conventional OLS uncertainty estimates and the HC3 covariance sensitivity check are reported in Section 3.2. Local logarithmic sensitivities and their relation to nominal effective tensile stress were evaluated as described in Section 3.3. These analyses apply to the actual combinations in Table 2 and constitute internal model assessment, not an independent experimental validation.

## 3. Results

### 3.1. Results of Repeated Tests and Lifetime Distributions

**Figure 2:**
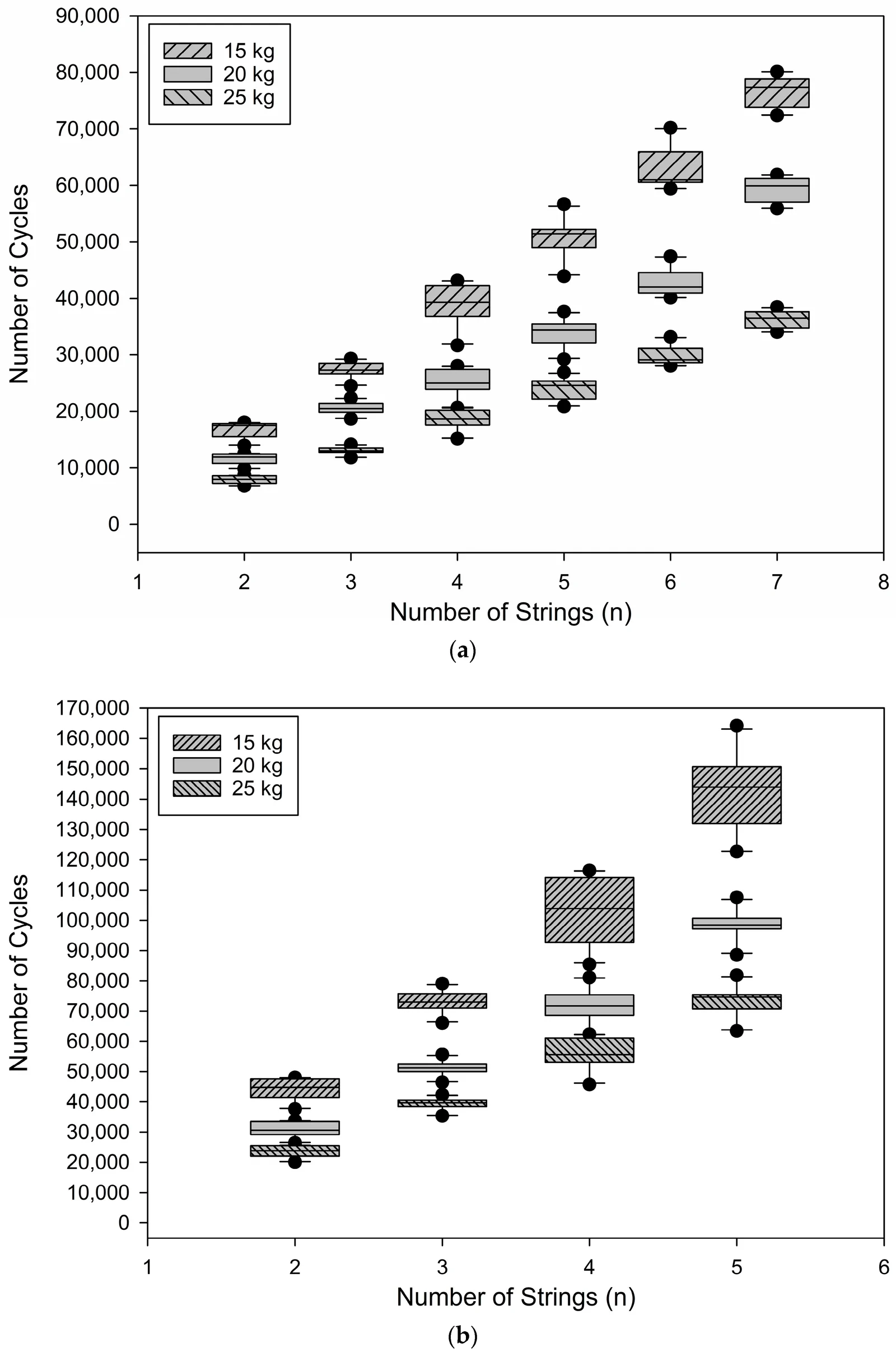

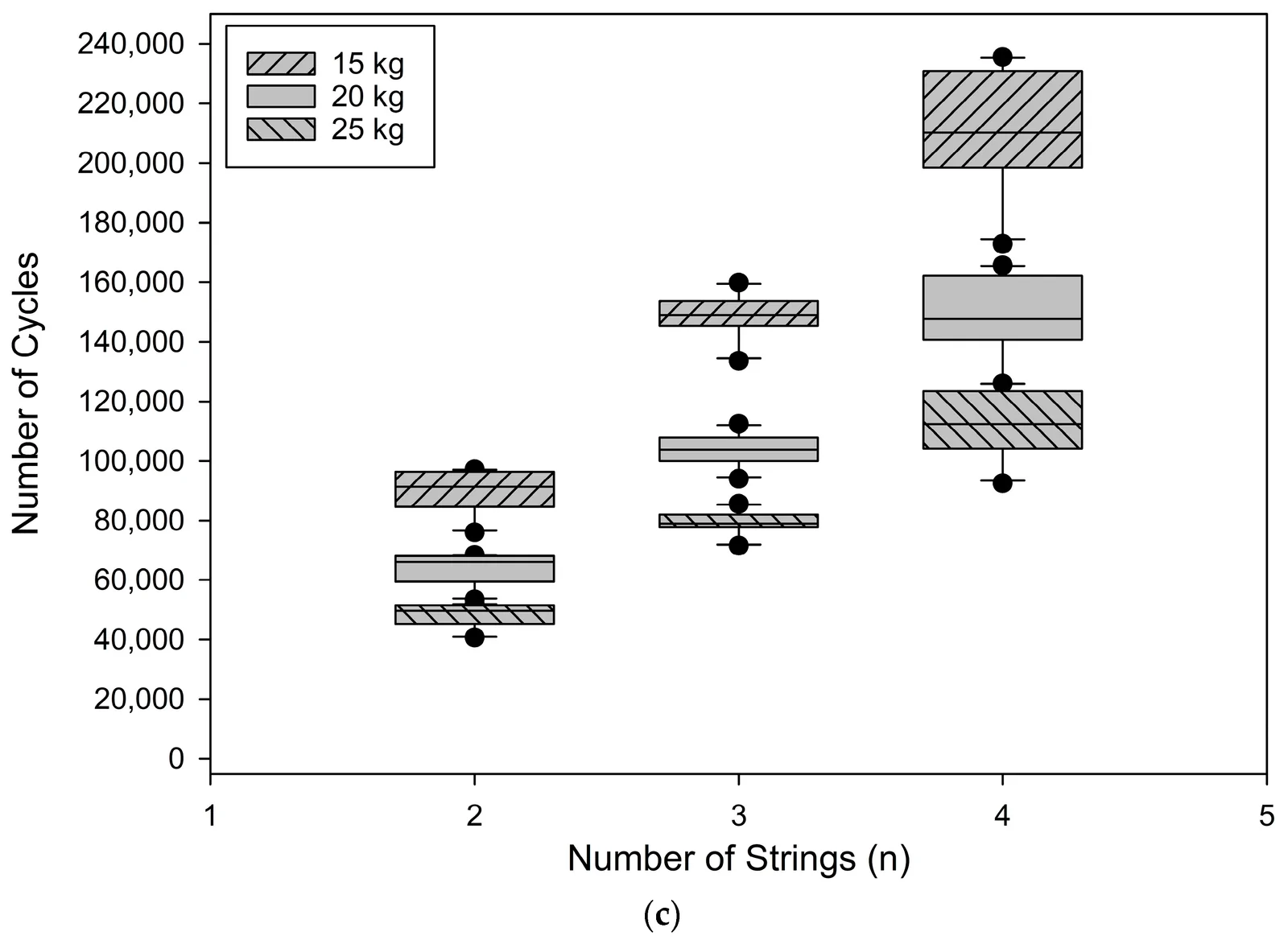
Effect of the number of strings and applied mass on the cyclic lifetime of the twisted string actuator for string diameters of (**a**) 1.0 mm, (**b**) 1.5 mm, and (**c**) 2.0 mm. The boxes represent the interquartile range (Q1–Q3), and the horizontal lines within the boxes indicate the medians. The whiskers extend to the most extreme data points within 1.5 times the interquartile range, while the circles indicate outliers beyond the whiskers.

Figure 2 presents the distributions of cyclic lifetime obtained from the repeated tests described in Section 2. The experimental matrix comprised 39 combinations of string diameter, string number, and applied mass, with ten repetitions per condition. The response of each specimen is the number of cycles to failure, *N_f_*, determined using the displacement-based termination criterion defined in Section 2. The box plots are used to compare the central tendency and within-condition spread across the tested configurations. For the 1.0 mm-diameter strings in Figure 2a, cyclic lifetime increased consistently as the number of strings increased from two to seven. At a given string number, the 15 kg condition exhibited the highest lifetime, followed by 20 kg and then 25 kg. The ordering of the medians was maintained across the configurations, indicating that the observed dependence on applied mass was reproduced throughout this diameter group. The 1.5 mm-diameter strings in Figure 2b showed the same ordering with applied mass and generally higher lifetimes than the 1.0 mm strings at common string-number and mass conditions. The four- and five-string configurations tested at 15 kg exhibited wider boxes and whisker ranges than the lower-lifetime configurations. Thus, the improvement in lifetime was accompanied by increased absolute scatter in these cases. The ordering of the median lifetimes remained distinguishable despite the wider distributions. For the 2.0 mm-diameter strings in Figure 2c, relatively high lifetimes were obtained even with two or three strings. At common values of string number and applied mass, the lifetime was greater than that of the smaller-diameter strings. The four-string configuration produced the highest-lifetime distributions within this diameter group and also exhibited a wider absolute spread. Overall, the repeated tests showed increasing cyclic lifetime with increasing string diameter and string number, and decreasing lifetime with increasing applied mass. These consistent trends motivate a multivariable description of the experimental response. The distributional comparisons establish the direction of the observed effects; they do not, by themselves, quantify contact pressure, load redistribution, or the contribution of a particular wear mechanism. Those mechanisms are considered through the nominal-stress interpretation and the post-failure observations in Section 3.3 and Section 3.4. The box plots illustrate the distributions and within-condition variability of the repeated lifetime measurements. Although ten measurements were obtained for each experimental condition, the present study focuses on predicting condition-wise mean lifetime rather than estimating specimen-level failure probabilities. Accordingly, Weibull and lognormal lifetime distributions were not independently fitted, and the results should not be interpreted as a probabilistic reliability assessment.

### 3.2. Second-Order Log-Polynomial Lifetime Model and Validation

A second-order log-polynomial formulation was fitted to the supplied condition-wise lifetime data to describe their dependence on string diameter, string number, and applied mass. A multiplicative power-law formulation has previously been used for TSA lifespan prediction [18]. That form was retained as a reduced comparison model, while the full second-order formulation included quadratic and interaction terms. Both models were recalculated from the same data and evaluated using the same validation protocol.

#### 3.2.1. Response Definition and Model Formulation

Consistent with the original condition-wise regression, let *N_f_*_,*ij*_ be the measured lifetime of repetition *j* under condition *i*, and let ${\bar{N}}_{f,i}$ be the arithmetic mean of the ten repetitions. The modeled response is the logarithm of that arithmetic mean, not the mean of the logarithms of the individual lifetimes:

The use of condition-wise arithmetic means reduces the influence of individual measurement fluctuations on the fitted response. However, this aggregation does not preserve the within-condition dispersion of the ten individual lifetime measurements in the regression model. Therefore, the proposed formulation predicts condition-wise mean lifetime rather than the probability distribution of individual specimen lifetimes.

(1) $${\bar{N}}_{f,i}=\frac{1}{10}\sum_{j=1}^{10}N_{f,\mathrm{ij}},\;\;y_{i}=\ln\left(\frac{{\bar{N}}_{f,i}}{N_{0}}\right),\;\;i=1,\ldots ,39$$

Here, *N*_0_ = 1 cycle is a reference value that makes the logarithm dimensionless. The following fixed reference conditions are adopted for the parameterization: *d*_0_ = 1.5 mm, *n*_0_ = 4, and *M*_0_ = 20 kg. These values define the coordinate origin and are not additional measurements. The normalized logarithmic predictors are

(2) $$x_{d,i}=\ln\left(\frac{d_{i}}{d_{0}}\right),\;\;x_{n,i}=\ln\left(\frac{n_{i}}{n_{0}}\right),\;\;x_{M,i}=\ln\left(\frac{M_{i}}{M_{0}}\right)$$

Using ***x_i_*** = (*x_d_*_,*i*_,*x_n_*_,*i*_,*x_M_*_,*i*_), the full second-order model is

(3) $$\begin{array}{c} y_{i}=\beta_{0}+\beta_{d}x_{d,i}+\beta_{n}x_{n,i}+\beta_{M}x_{M,i}\\ +\beta_{\mathrm{dd}}x_{d,i}^{2}+\beta_{\mathrm{nn}}x_{n,i}^{2}+\beta_{\mathrm{MM}}x_{M,i}^{2}\\ +\beta_{\mathrm{dn}}x_{d,i}x_{n,i}+\beta_{\mathrm{dM}}x_{d,i}x_{M,i}+\beta_{\mathrm{nM}}x_{n,i}x_{M,i}+\varepsilon_{i} \end{array}$$

The right-hand side excluding *ε_i_* defines *f*_2_(***x_i_***; ***β***). The ten coefficients comprise one intercept, three first-order coefficients, three pure quadratic coefficients, and three interaction coefficients; *ε_i_* is the log-scale residual. All ten coefficients were estimated jointly, with no higher-order terms removed. In particular, the first-order coefficients are not fixed at the exponents of the previously reported power-law model. Setting the six higher-order coefficients to zero recovers the reduced power-law form in the same normalized coordinates.

The direct back-transformation of the fitted response is

(4) $${\hat{\bar{N}}}_{f,i}=N_{0}\exp\left[f_{2}\left(x_{i};\hat{\beta }\right)\right]$$

Both models were back-transformed directly by exponentiation, without a smearing factor or other bias correction. This procedure gives positive fitted lifetimes but does not guarantee an unbiased conditional arithmetic mean. Original-cycle-scale errors therefore quantify the performance of this stated transformation, rather than an assumed bias-corrected predictor.

#### 3.2.2. Coefficient Estimation and Condition-Wise Validation

The full coefficient vector was estimated by ordinary least squares from the 39 logarithmic condition means in Equation (1). Multicollinearity was assessed using the 39 tested configurations and the normalized logarithmic predictors defined in Section 3.2.1. The Pearson correlation between *x_d_* and *x_n_* was −0.373, whereas the correlations of *x_M_* with both *x_d_* and *x_n_* were approximately zero. Variance inflation factors were calculated for all nine non-intercept terms of the full second-order model, including the squared and interaction terms, using auxiliary regressions with an intercept. The VIFs ranged from 1.27 to 4.39, with the largest value associated with *x_n_*. These diagnostics quantify the dependence among the model terms; they do not remove the limitations imposed by the incomplete factorial design. Table 3 gives the estimates and two-sided 95% t-based OLS intervals. These intervals assume independent, homoscedastic log-scale errors and a correctly specified mean function. They describe model-conditional uncertainty, not verified independence of the experimental records.

Intervals use 29 residual degrees of freedom and the conventional OLS covariance matrix. They are not multiplicity-adjusted. All ten terms were retained. The parameter estimates and intervals must be recalculated if any source values or condition labels change. Predictive evaluation used leave-one-condition-out cross-validation. In each of 39 folds, both models were refitted to the remaining 38 condition means and used to predict the excluded mean. No individual record from the excluded condition entered the fit. No term selection, tuning, or inverse-transform correction was performed. This is internal condition-wise validation; it does not establish independence where values may be related across conditions. For *K* evaluated conditions, error measures in the original cycle scale are defined as

(5) $$\begin{array}{c} \mathrm{MAPE}=\frac{100}{K}\sum_{i=1}^{K}\left|\frac{{\hat{\bar{N}}}_{f,i}-{\bar{N}}_{f,i}}{{\bar{N}}_{f,i}}\right|\\ \mathrm{RMSE}=\sqrt{\frac{1}{K}\sum_{i=1}^{K}{\left({\hat{\bar{N}}}_{f,i}-{\bar{N}}_{f,i}\right)}^{2}} \end{array}$$

For cross-validation, the predictions in Equation (5) are the held-out predictions and *K* = 39. In-sample and held-out results are reported separately. Log-scale fit statistics are distinguished from cycle-scale prediction statistics. The assessment evaluates held-out predictions for the 39 tested combinations in Table 2, with each condition mean predicted from a model fitted to the other 38 conditions. This constitutes internal cross-validation, not validation against an independently acquired experimental dataset. The test matrix defines the scope of the analyzed data, not independently validated limits of model applicability. Predictive performance at untested combinations or under changes in string material, effective free length, contraction displacement, or motor speed has not been established by this analysis.

#### 3.2.3. Model Comparison and Numerical Results

Table 4 compares matched fits of the four-coefficient reduced power-law model and the ten-coefficient second-order model. The comparison distinguishes log-scale fit statistics from original-cycle-scale prediction errors. The full second-order model is carried forward for exploratory sensitivity analysis, while the comparison model is retained to show the effect of added complexity.

**Figure 3:**
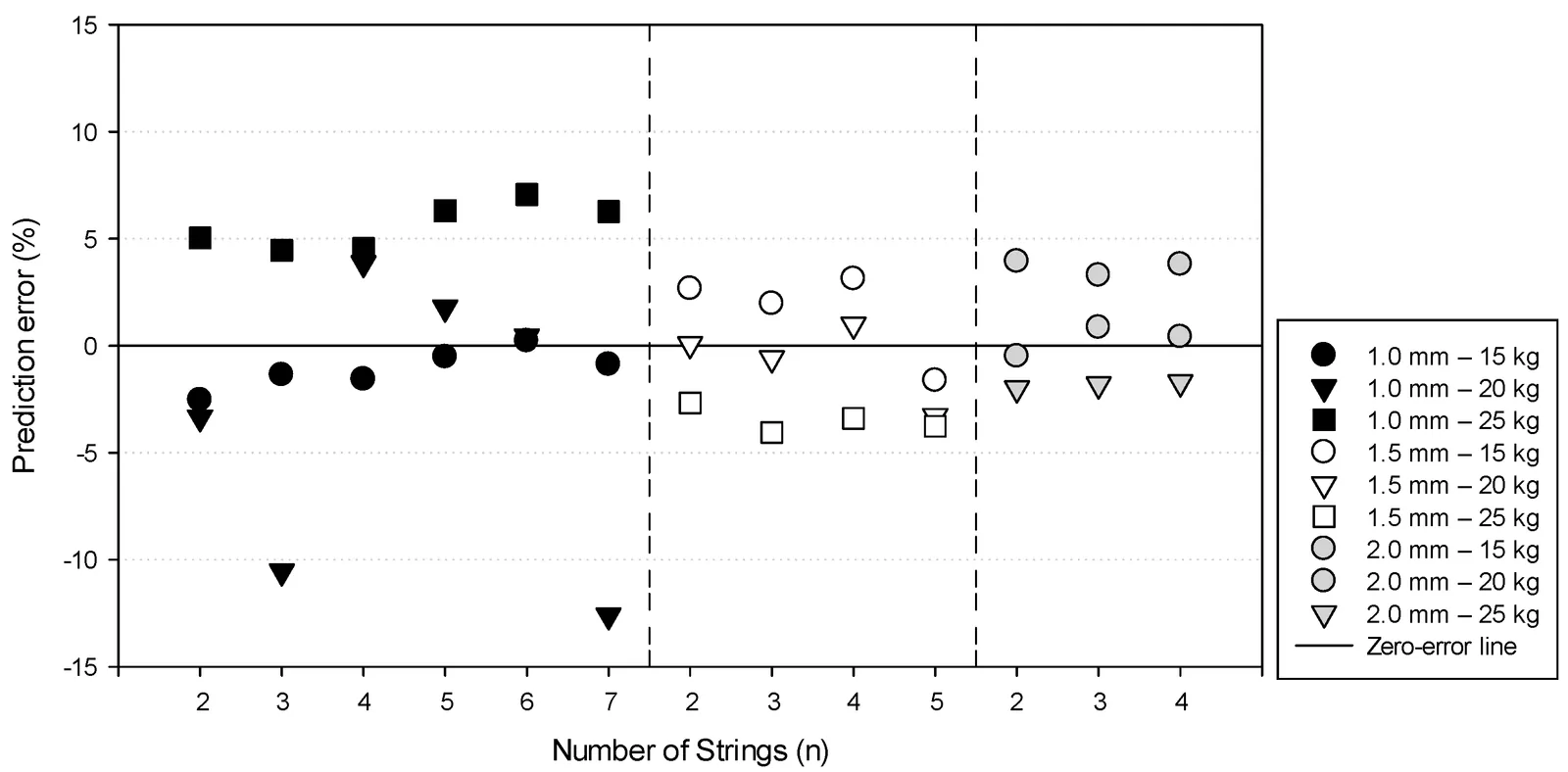
Signed percentage prediction errors of the second-order log-polynomial lifetime model obtained using leave-one-condition-out cross-validation (LOOCV) for UHMWPE twisted string actuators with nominal diameters of 1.0, 1.5, and 2.0 mm. The prediction error is defined as 100(*N*_pred_ − *N*_exp_)/*N*_exp_, where positive and negative values indicate overprediction and underprediction, respectively. The horizontal axis indicates the number of strings, while different marker shapes distinguish the applied masses of 15, 20, and 25 kg. The horizontal solid line represents zero prediction error, and the vertical dashed lines separate the string-diameter groups.

All statistics were recalculated from the same 39 responses using direct exponentiation. For clarity, the reported regression coefficients and prediction-performance metrics were rounded to an appropriate number of decimal places, while full numerical precision was retained in all calculations. The adjusted R^2^ values refer to the log-scale fits. AICc and BIC use Gaussian log-residual likelihoods and count the residual variance as one additional parameter (k = 5 and 11). These remain conditional model comparisons. The second-order model gave an in-sample MAPE of 2.1212% and an RMSE of 1766.16 cycles. Its condition-wise cross-validated MAPE was 2.8274%, compared with 3.4433% for the reduced model, a relative decrease of 17.89%. The cross-validated RMSE decreased from 2748.15 to 2357.23 cycles. However, the maximum held-out absolute percentage error increased from 13.9588% to 14.2056%; improvement was not uniform across conditions. AICc was slightly lower for the second-order model, whereas BIC was slightly lower for the reduced model. Thus, the aggregate errors favor the second-order fit on this dataset, but the criteria do not establish an unqualified advantage. Under conventional OLS intervals, the mass-curvature and diameter–mass interaction coefficients excluded zero. The remaining four higher-order intervals included zero. An HC3 covariance sensitivity check gave a two-sided t-based *p* value of 0.131 for mass curvature and 0.000038 for the diameter–mass interaction, compared with conventional OLS *p* values of 0.0339 and 0.00210. Accordingly, the evidence for mass curvature depends on the covariance assumption. Neither calculation accounts for unknown dependence between source records. The numerical fit does not certify the provenance of the data or establish a causal friction mechanism. Figure 3 presents the signed percentage prediction errors of the second-order log-polynomial model obtained through leave-one-condition-out cross-validation for the 39 tested conditions.

### 3.3. Effective-Stress Interpretation and Local Sensitivity Analysis

The nominal effective tensile-stress index is retained to relate the geometric and loading variables to a common mechanical quantity. For a bundle of *n* strings with individual nominal diameter *d*, the nominal total cross-sectional area is *A_t_* = *nπd*^2^/4. The corresponding index is

(6) $$\sigma_{\mathrm{eff}}=\frac{\mathrm{Mg}}{A_{t}}=\frac{4\mathrm{Mg}}{\pi nd^{2}}$$

Here, *g* denotes gravitational acceleration. When *M* is expressed in kilograms and *d* in millimetres, the resulting index is in N/mm^2^, or MPa. The expression combines the applied mass with the nominal bundle area; it does not resolve internal voids, helical filament orientations, or the spatial distribution of load among contacting strings.

Accordingly, *σ*_eff_ is not interpreted as the actual stress on an individual filament. Shah and Sockalingam [29] reported that sharp transverse contact geometries can induce progressive filament failure and reduce the load-carrying capacity of Dyneema^®^ SK-76 yarns (DSM Dyneema, Geleen, the Netherlands). Although their quasi-static transverse loading differs from cyclic TSA operation, this observation supports distinguishing local contact-induced loading from a nominal axial stress index.

With $\sigma_{0}=4M_{0}g/\left(\pi n_{0}d_{0}^{2}\right)$ defined using the same reference condition as Equation (2), the normalized logarithmic stress is

(7) $$s=\ln\left(\frac{\sigma_{\mathrm{eff}}}{\sigma_{0}}\right)=x_{M}-x_{n}-2x_{d}$$

Instead of assigning constant exponents to the second-order formulation, its local logarithmic sensitivities are obtained directly by differentiation. With $\hat{y}=f_{2}\left(x;\hat{\beta }\right)$,

(8) $$\begin{array}{c} S_{d}=\frac{\partial \hat{y}}{\partial \ln d}={\hat{\beta }}_{d}+2{\hat{\beta }}_{\mathrm{dd}}x_{d}+{\hat{\beta }}_{\mathrm{dn}}x_{n}+{\hat{\beta }}_{\mathrm{dM}}x_{M}\\ S_{n}=\frac{\partial \hat{y}}{\partial \ln n}={\hat{\beta }}_{n}+2{\hat{\beta }}_{\mathrm{nn}}x_{n}+{\hat{\beta }}_{\mathrm{dn}}x_{d}+{\hat{\beta }}_{\mathrm{nM}}x_{M}\\ S_{M}=\frac{\partial \hat{y}}{\partial \ln M}={\hat{\beta }}_{M}+2{\hat{\beta }}_{\mathrm{MM}}x_{M}+{\hat{\beta }}_{\mathrm{dM}}x_{d}+{\hat{\beta }}_{\mathrm{nM}}x_{n} \end{array}$$

At the reference condition, all three normalized predictors are zero, so the local sensitivities reduce to the fitted first-order coefficients of the second-order model. Away from that condition, the fitted quadratic and interaction coefficients permit sensitivity to vary with geometry and loading. For small proportional changes, each sensitivity describes the corresponding proportional change in the directly back-transformed fitted response, with the other predictors held fixed.

Because string number is discrete, *S_n_* describes a derivative of the continuous fitted surface, not a physically realizable infinitesimal addition of a string. Design comparisons should therefore also evaluate predictions at the supported integer configurations. All local interpretations are limited to the region supported by the actual test combinations.

Stress-equivalent local sensitivities provide a direct comparison between the three variable effects:

(9) $$m_{d}\left(x\right)=\frac{S_{d}\left(x\right)}{2},\;\;m_{n}\left(x\right)=S_{n}\left(x\right),\;\;m_{M}\left(x\right)=-S_{M}\left(x\right).$$

For a fitted log-lifetime response that depends only on *s* in Equation (7), the chain rule requires these three quantities to coincide at a given condition. This remains true when the stress–life slope varies with stress. Consequently, statistically resolved differences between these quantities would indicate configuration dependence beyond that single nominal-stress index. Agreement at the sampled conditions, however, would not independently prove a universal stress–life law. Quadratic or interaction coefficients alone do not establish a departure from stress-only behavior. A curved relationship expressed only in *s* = *x_M_* − *x_n_* − 2*x_d_* can also produce quadratic and interaction terms when expanded in *x_d_*, *x_n_*, and *x_M_*. The assessment therefore concerns the fitted sensitivities and their joint uncertainty, rather than assigning a separate physical mechanism to every higher-order term. At the reference condition (d = 1.5 mm, n = 4, M = 20 kg), the local logarithmic sensitivities were 2.527175 for diameter, 1.258140 for string number, and −1.322205 for applied mass. Across the 39 tested combinations, their ranges were 2.315050–2.729428, 1.150868–1.291037, and −1.666717 to −0.962697, respectively. These are extrema at the tested combinations, not confidence limits or bounds over an untested rectangular domain. The corresponding stress-equivalent values at the reference condition were 1.263588, 1.258140, and 1.322205. Using the full OLS coefficient covariance, a joint test of their equality gave F(2, 29) = 1.0244 and *p* = 0.3716. Equality was therefore not rejected at that reference condition under the stated model assumptions. This local comparison neither establishes equality throughout the test domain nor independently validates a stress-only lifetime law. The approximate value of 1.27 was derived from the coefficients of the reduced power-law model fitted to the present experimental data, rather than adopted from a previous publication. This value was not imposed as a fixed exponent in the second-order model. The second-order sensitivities are conditional descriptors of the supplied data and must be re-evaluated if condition labels or source values are corrected.

These sensitivities characterize the statistical response surface; they are not measurements of inter-string contact pressure, sliding distance, or local filament stress. Surface damage and fracture morphology provide qualitative context for interpretation but cannot identify a unique physical cause for an individual regression coefficient. The observations in Section 3.4 are therefore kept separate from the numerical evidence required for the model.

### 3.4. Post-Failure Morphology and Qualitative Damage Observations

**Figure 4:**
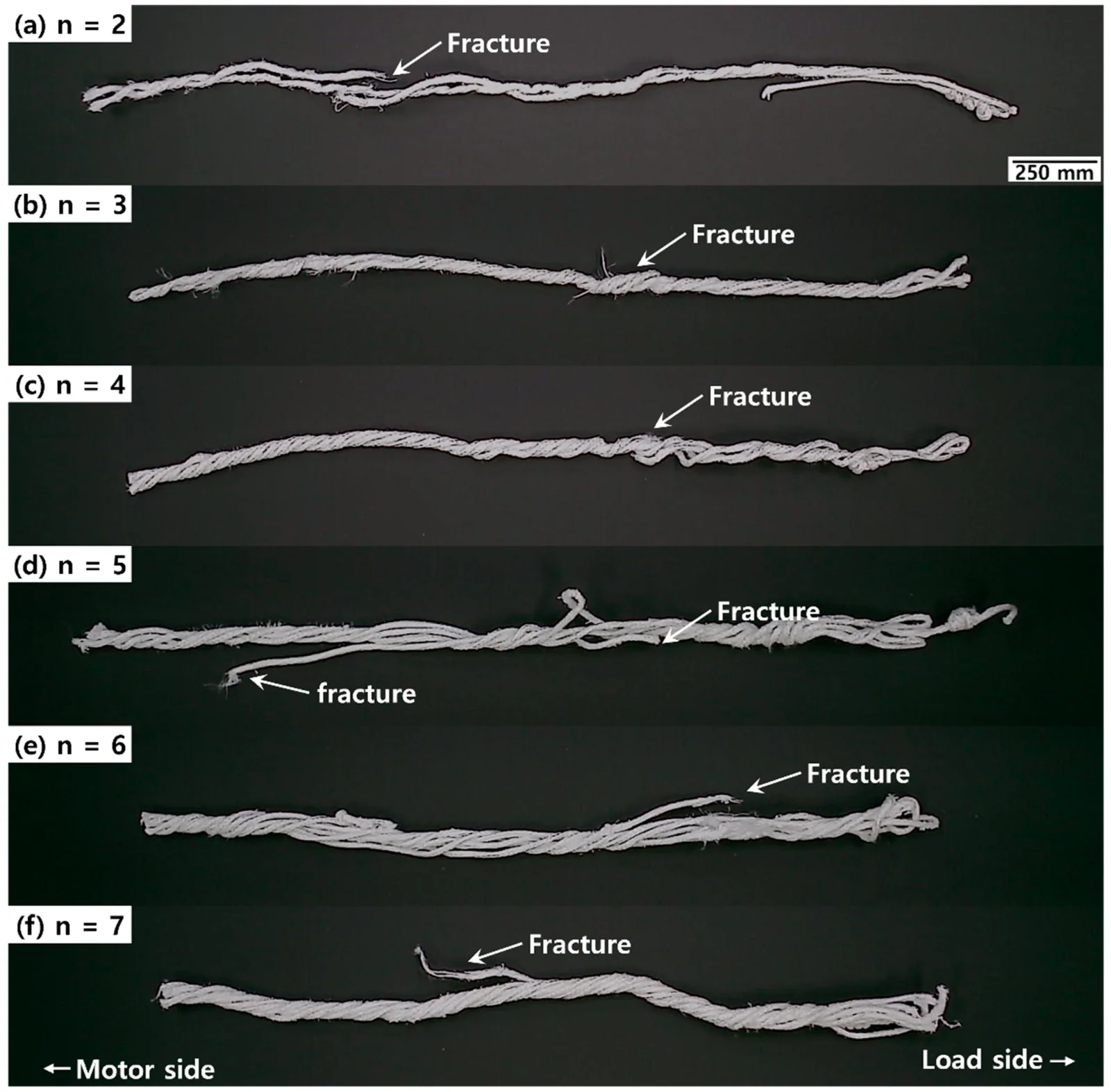
Selected post-test morphologies of 1.0 mm diameter UHMWPE strings with two to seven strings. These images are provided to illustrate characteristic fracture features rather than to establish diameter-dependent differences in failure morphology: (**a**) *n* = 2, (**b**) *n* = 3, (**c**) *n* = 4, (**d**) *n* = 5, (**e**) *n* = 6, and (**f**) *n* = 7. The arrows indicate the identified fracture locations. All specimens are oriented from the motor side to the load side.

Figure 4 presents selected post-test morphologies of 1.0 mm-diameter UHMWPE strings with two to seven strings. These specimens were used to illustrate representative fracture characteristics under repeated twisting–untwisting operation. The observations were qualitative and were not intended to establish differences in fracture mechanisms among the three nominal diameters. Since scanning electron microscopy was not performed, the analysis was limited to the surface and fracture features visible in the available optical observations. The specimens are oriented consistently from the motor side to the load side, and the arrows identify visible fracture locations. For the smaller bundles, separation between individual strings and localized rupture sites is relatively distinct. Larger bundles exhibit outward displacement of individual strings and damage at multiple locations. Two localized fracture sites can be identified in the illustrated five-string configuration.

In the illustrated specimens, visible fracture regions are mainly located within the active string length. However, these representative observations do not establish the statistical distribution of fracture locations across all tested specimens or demonstrate that the same fracture patterns occur for the 1.5 and 2.0 mm strings. This distribution is compatible with localized damage during repeated twisting and inter-string contact. Nevertheless, the illustrated examples do not establish a statistical distribution of failure locations or demonstrate the effectiveness of the end reinforcement independently. Multiple post-test fracture sites are consistent with possible load redistribution, but they do not establish the temporal sequence of string failures or quantify the redistributed load.

**Figure 5:**
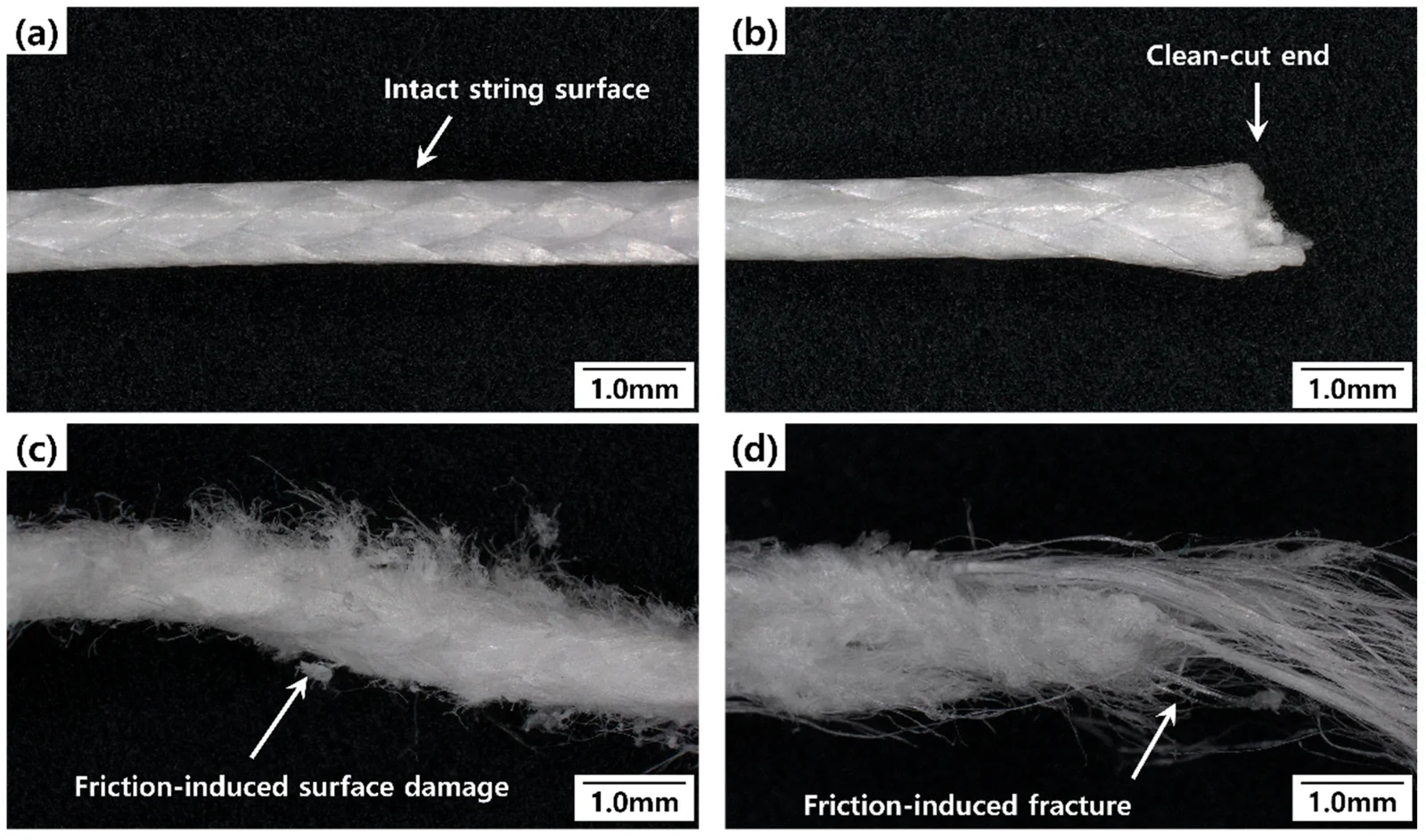
Representative optical images of UHMWPE string surfaces and failure morphology: (**a**) intact string surface, (**b**) artificially cut end, (**c**) surface damage after repeated twisting–untwisting cycles, and (**d**) fracture region showing filament fibrillation and pull-out.

Figure 5 compares the intact string surface, an artificially cut end, a surface region damaged after repeated actuation, and a final fracture region. The intact string exhibits a relatively regular braided surface without conspicuous loosening. The artificially cut reference has a localized cut boundary. In contrast, the repeatedly actuated surface displays fuzzing, exposed filaments, and partial filament breakage. Extensive filament pull-out and irregular filament separation are visible near the final fracture region, distinguishing it from the artificially cut reference. Taken together with the imposed twist–untwist loading history, these features are consistent with progressive filament damage associated with repeated contact and relative sliding. A reduction in the number of intact load-bearing filaments could increase the load carried by the surviving filaments and thereby provide a qualitative connection to the nominal-stress interpretation. The observed fuzzing, filament exposure, partial breakage, and pull-out are therefore relevant to understanding how repeated actuation can degrade the load-bearing structure. The post-failure observations provide a qualitative description of damage under the imposed twist–untwist loading history. No quantitative morphological damage metric was obtained or statistically related to the measured lifetimes or fitted coefficients. Detailed cross-sectional examination of the fractured strings was not performed because the post-failure analysis was designed to provide qualitative observations of surface damage and visible fracture morphology rather than quantitative characterization of internal filament damage. Future investigations could employ scanning electron microscopy (SEM) of fracture surfaces and optical or electron microscopy of prepared transverse sections to examine filament rupture, pull-out, and changes in the remaining load-bearing structure. Quantitative morphological parameters, such as the fraction of damaged filaments or the remaining intact cross-sectional area, could then be compared with the measured cycles to failure under different string configurations and applied loads. Although a relationship between cross-sectional damage and cyclic lifetime is mechanically plausible, it was not directly established in the present study. The proposed sequence from surface damage to load redistribution and final rupture therefore remains an interpretation, rather than a directly recorded history of damage development. Neither the change in load-bearing area nor the local load redistribution was quantified. In addition, local temperature and inter-string contact pressure were not measured during the experiments. Therefore, the observed surface damage cannot be directly attributed to a specific thermal or mechanical mechanism, and the commanded motor speed of 1500 rpm alone does not demonstrate that the UHMWPE strings reached their melting temperature. Accordingly, these observations provide context for discussing possible damage processes; they do not constitute quantitative validation of the lifetime model or identify the physical origin of its coefficients.

## 4. Discussion

### 4.1. Physical Interpretation of the Second-Order Model and Effective-Stress Scaling

The second-order formulation in Section 3.2 extends the reduced power-law description by allowing the logarithmic sensitivities to vary with string configuration and applied mass. Its role is not simply to replace three constant exponents with a longer equation, but to describe curvature and cross-variable dependence that can be evaluated against the observed condition means. The reduced model remains a comparison baseline. Neither formulation constitutes a constitutive law for UHMWPE fibers, and the fitted coefficients should be interpreted at the actuator-system level under the test conditions used here. The matched comparison in Table 4 shows a reduction in condition-wise cross-validated MAPE from 3.4433% to 2.8274% and in RMSE from 2748.15 to 2357.23 cycles. The 17.89% relative reduction in mean percentage error supports carrying the second-order surface forward for exploratory sensitivity analysis on this dataset. However, the maximum held-out error increased slightly, from 13.9588% to 14.2056%, and the small AICc and BIC differences did not favor the same model. Consequently, the additional terms improve average internal prediction without establishing uniform improvement across all configurations or an unconditional advantage over the simpler model. The nominal effective tensile-stress index in Section 3.3 provides a mechanical reference for interpreting the fitted surface. Increasing nominal diameter or string number increases the nominal bundle area, whereas increasing applied mass raises the nominal tensile load. At the reference condition of 1.5 mm, four strings, and 20 kg, the stress-equivalent local sensitivities were 1.263588, 1.258140, and 1.322205 for the diameter, string-number, and mass directions, respectively. The joint OLS-based comparison did not reject their equality at that condition (F(2, 29) = 1.0244, *p* = 0.3716). This result is compatible with a local nominal-stress interpretation, but it neither proves equality throughout the test domain nor independently validates a universal stress–life law. In particular, the presence of quadratic and interaction terms is not, by itself, evidence that nominal-stress scaling has failed. A curved relationship between log lifetime and log nominal stress can generate such terms when expressed in the three design variables. The relevant comparison therefore concerns the stress-equivalent sensitivities and their joint uncertainty, rather than the number of terms in the model. The characteristic value near 1.27 from the reduced model is not imposed on the second-order surface and should not be interpreted as a constant exponent governing all of its predictions. The distinction between nominal loading and the evolving contact configuration is mechanically relevant for TSAs. Xu et al. [30] incorporated string elongation and load-dependent geometric changes into a compliant TSA model with continuously variable transmission. Usman et al. [17] reported load- and string-configuration-dependent cyclic lifetimes, while Sadachika et al. [31] investigated repetitive twisting and residual-strength degradation in synthetic-fiber ropes. These studies provide context for interpreting configuration-sensitive behavior, but they do not validate the numerical coefficients fitted here. Previous studies on HMPE ropes have investigated dynamic tensile fatigue [19] and cyclic bend-over-sheave loading [32]. However, these loading conditions differ substantially from the repeated twisting–untwisting motion investigated in the present study. Therefore, the relatively shallow stress-equivalent sensitivity observed here cannot be directly compared with conventional rope fatigue exponents. Frictional wear, inter-string contact, and time-dependent deformation may contribute to the observed lifetime behavior, although their individual contributions were not independently quantified. Within these limits, the response surface and nominal-stress index have complementary roles: the former predicts condition-dependent lifetime trends, whereas the latter organizes their physical interpretation. Possible effects of load sharing, local bending, contact, and sliding are considered below as hypotheses consistent with the observations, not as separately measured contributions. The statistical intervals and internal validation remain conditional on the response assignments and assumptions described in Section 3.

### 4.2. Effect of String Diameter

The local logarithmic diameter sensitivity, *S_d_*, was positive at all 39 evaluated combinations. Its value was 2.527175 at the reference condition and ranged from 2.315050 to 2.729428 across the tested combinations. Thus, the fitted surface predicts an increase in lifetime with diameter at those evaluated points. Among the three local sensitivities, the diameter sensitivity had the largest absolute magnitude over these points; this is a comparison of proportional changes in the predictors, not a ranking of the variance contributions of the experimental factors. For a sufficiently small diameter change near the reference condition, a 1% increase corresponds to an approximately 2.53% increase in the fitted lifetime, with string number and applied mass held fixed. Substitution of the coefficients in Table 3 into the diameter derivative defined in Section 3.3 gives

*S_d_* = 2.527175 − 0.197818 *x_d_* + 0.075081 *x_n_* + 0.358641 *x_M_*

This expression makes clear why the original constant diameter exponent cannot be used as the governing coefficient of the second-order model. The positive diameter–mass interaction means that, at fixed diameter and string number, the modeled proportional benefit of increasing diameter becomes larger as applied mass increases. The interval for this interaction excluded zero under conventional OLS, and its association remained supported in the reported HC3 sensitivity check. In contrast, the interval for the pure diameter-squared term included zero. The negative fitted diameter-curvature coefficient therefore does not, on its own, establish a reproducible diminishing-return mechanism. The positive diameter effect is consistent with the reduction in nominal tensile stress associated with increasing nominal load-bearing area. However, diameter also changes the geometry of the twisted bundle under the initial 50 mm contraction setting. The twist excursion required to achieve that displacement, local curvature, and inter-string sliding may consequently vary with diameter. These quantities were not quantified as independent explanatory variables in the lifetime regression, so the diameter–mass interaction cannot be assigned uniquely to any one of them. Ali et al. [33] evaluated TSA lifetime at different twisting amplitudes and reported shorter lifetimes at higher contraction levels in their actuator configuration. Their results support treating the imposed twisting range as part of the operating definition of a TSA durability test. Accordingly, the local diameter sensitivity describes variation in the fitted lifetime response across the tested commercial string sizes, with string number and applied mass held fixed in the model. It should not be interpreted as the effect of changing geometric diameter while holding all constituent-yarn and braid parameters constant. Potential differences in yarn count, constituent-yarn linear density, and braid pitch were not resolved independently and may contribute to the observed size dependence and diameter–mass association. The fitted coefficients are therefore descriptors of the tested product configurations and operating conditions, rather than isolated geometric effects or intrinsic material constants. Predictions for other string constructions or untested configurations require additional validation.

### 4.3. Effect of String Number and Load Sharing

The local logarithmic sensitivity to string number, *S_n_*, was 1.258140 at the reference condition and ranged from 1.150868 to 1.291037 at the 39 tested combinations. All evaluated values were positive, consistent with the observed increase in cyclic lifetime with string number. The fitted derivative is

*S_n_* = 1.258140 + 0.106920 *x_n_* + 0.075081 *x_d_* − 0.012185 *x_M_*

The relatively narrow range indicates that the fitted proportional string-number effect varies less over the evaluated configurations than the mass sensitivity. However, the conventional confidence intervals for the string-number-squared, diameter–string-number, and string-number–mass terms all included zero. The current fit therefore describes possible configuration dependence without providing strong evidence for each of these individual higher-order effects. In particular, it does not justify a general statement that each additional string becomes systematically more or less effective. Because string number is discrete, *S_n_* is a derivative of the continuous response surface, not an experimentally realizable infinitesimal addition of a string. A finite change in string count must be evaluated using the model predictions at both supported configurations. For example, evaluating the fitted surface at 1.5 mm and 20 kg gives approximately 31,878.79 cycles for two strings and 74,316.46 cycles for four strings, a predicted ratio of 2.33. These are fitted values, not additional measurements, and that ratio applies to this particular comparison rather than constituting a universal doubling factor. The positive string-number effect is consistent with distributing the nominal external load over a larger bundle area. At the same time, additional strings alter the bundle arrangement and the possible contact interfaces. Differences in initial tension, effective length, and alignment may produce nonuniform local loading, while contact and relative sliding can contribute to damage. The post-test example with five strings contains more than one visible fracture location, which is compatible with redistribution following localized damage. It does not establish the order of the failures, quantify the redistributed load, or show how much structural redundancy increased the measured lifetime. The wider absolute scatter observed for some higher-lifetime configurations should likewise not be assigned to load sharing alone. The fitted string-number sensitivity is therefore best treated as a system-level descriptor of the observed bundle response, with uniform preparation and controlled alignment remaining important for meaningful comparisons.

### 4.4. Effect of Applied Mass and Frictional Damage

The local logarithmic mass sensitivity, *S_M_*, was negative at every tested combination. It was −1.322205 at the reference condition and ranged from −1.666717 to −0.962697 across the evaluated combinations. These signs are consistent with shorter lifetimes under higher applied mass. Near the reference condition, a sufficiently small 1% increase in mass corresponds to an approximately 1.32% decrease in fitted lifetime, but that proportional rate is not constant throughout the second-order surface. The fitted derivative is

*S_M_* = −1.322205 − 0.861673 *x_M_* + 0.358641 *x_d_* − 0.012185 *x_n_*

The positive diameter–mass coefficient indicates that increasing diameter makes the mass sensitivity less negative when mass and string number are otherwise fixed. Equivalently, the modeled effect of increasing mass is more adverse for thinner strings within the evaluated region. This is consistent with nominal area influencing the severity of external loading. It is a statistical cross-dependence of the fitted response, not a direct measurement of contact pressure, friction coefficient, or load concentration. The negative pure mass-curvature coefficient makes the fitted mass sensitivity more negative as mass increases. Its inferential support, however, was sensitive to the covariance treatment: the conventional OLS *p* value was 0.0339, whereas the reported HC3 value was 0.131. The corresponding diameter–mass interaction remained supported under both calculations. These results favor a cautious distinction between the clear negative mass dependence, the better-supported diameter–mass association, and the less stable evidence for pure mass curvature. None of these covariance calculations resolves unknown dependence among the source records. Finite load changes must therefore be calculated from the full surface rather than from a single expression such as a fixed mass ratio raised to a constant exponent. For example, at four strings, the model predicts that increasing applied mass from 15 to 25 kg leaves approximately 47.93%, 51.62%, and 54.42% of the 15 kg fitted lifetime for diameters of 1.0, 1.5, and 2.0 mm, respectively. These comparisons use combinations represented in Table 2 and are calculations from the fitted model, not additional experimental results. They illustrate why one universal remaining-life percentage is not appropriate for the second-order formulation. Higher nominal loading provides a plausible route to more severe damage when it acts together with repeated contact and sliding. As surface filaments become damaged, a smaller intact load-bearing structure may carry the external force, potentially increasing local stresses. Usman et al. [17] reported a decrease in TSA lifetime with increased applied mass. Repetitive twisting was investigated by Sadachika et al. [31], while Horigome and Endo [34] examined repetitive bending durability. These studies provide complementary context, but differences in deformation mode, materials, and test settings prevent their results from identifying the physical origin of the present interaction coefficient. Accordingly, the surface fuzzing and filament damage described in Section 3.4 support a friction-associated interpretation of the lifetime trends, while the specific contribution of contact pressure, sliding distance, and heat generation remains unresolved. Direct measurements of those quantities would be required to separate a nominal tensile-load effect from additional tribological effects. The statistical model should not be interpreted as such a separation by itself. An additional limitation is that elapsed time under load was not evaluated as an independent explanatory variable in the present lifetime analysis. The 300 s pre-loading period does not exclude subsequent creep-related deformation or a possible contribution of creep rupture. The commanded motor speed of 1500 rpm specifies shaft rotation rather than the twist–untwist cycle frequency; the duration of a cycle also depends on the rotation required to reach the target contraction and on the reversal sequence. No test-resolved analysis of accumulated elongation or changes in the turns required to achieve the prescribed 50 mm contraction is presented here. Consequently, the current analysis cannot determine whether failure is primarily cycle-controlled, time-controlled, or governed by their combined effects. The fitted model describes cycles to the defined endpoint under the prescribed actuation protocol, rather than establishing a rate-independent fatigue law. Resolving these contributions requires time-resolved deformation and rotation records and experimental comparisons that distinguish repeated actuation from sustained loading.

### 4.5. Failure Mechanism

The observations in Section 3.4 provide qualitative evidence relevant to the physical interpretation of the second-order model. Compared with the unused surface, the repeatedly actuated strings show fuzzing, filament exposure, partial breakage, and extensive pull-out near the fracture region. The artificially cut reference has a more localized cut boundary. Considered together with the imposed twist–untwist loading history, these features are compatible with accumulated filament damage associated with repeated contact and relative sliding. They do not, on their own, distinguish every possible contribution of wear, tensile loading, local bending, and twisting. A plausible damage sequence begins with abrasion and loosening of exposed surface filaments during repeated actuation. Continued damage may reduce the intact load-bearing area, after which the remaining filaments and strings carry a greater local share of the external force. This redistribution can provide a feedback between damage and local stress concentration, eventually contributing to final rupture. The sequence is a mechanistic interpretation rather than a directly recorded chronology: the images were acquired after testing and neither the evolving area nor the local redistribution was quantified. The final rupture may still occur rapidly after a period of accumulated damage. Repetitive-twisting studies of synthetic-fiber ropes provide related evidence of residual-strength changes [31]. Sliding wear and performance degradation have also been investigated in polyurethane-coated HMPE ropes in sheave systems [23], while repeated bend-over-sheave loading and torsion have been examined for braided HMPE ropes [32,35]. These references motivate consideration of coupled loading and contact effects. They do not establish that the same damage rate or a unique wear mechanism applies to the present TSA, and they are not independent validations of the fitted quadratic coefficients. The representative images show fracture locations within the active string length; however, these observations do not establish the overall distribution of fracture locations across all tested specimens. Since a complete specimen-wise classification of fracture locations was not included in the present analysis, no quantitative conclusion is drawn regarding the frequency of failures at or near the knots. The heat-shrink reinforcement and universal coupling were intended to limit unwanted end effects; their contribution to lifetime was not isolated by a separate comparison. Horigome et al. [36] developed a terminal fixation method for synthetic-fiber ropes and evaluated its fixation strength. That study supports attention to end fixation, not a quantitative conclusion that the present reinforcement increased cyclic lifetime. The distinction between a lifetime model and an evolving-damage model is important. The fitted surface predicts an end-of-life response from initial configuration and applied mass; it does not contain a measured history of filament loss, contact force, or temperature. The local sensitivities quantify how the fitted terminal lifetime changes with the selected design variables. Neither a nonzero interaction coefficient nor agreement between stress-equivalent sensitivities identifies a unique damage mechanism. Similarly, the observed images do not demonstrate local melting or thermal degradation without temperature measurements or evidence of thermally altered filament structure. Taken together, the recorded lifetime trends, internal model comparison, and post-failure observations support using the second-order formulation as a conditional engineering description of this test series. Its application should remain restricted to the supported combinations and the same material, 250 mm jig-to-jig distance, initial 50 mm contraction setting, and 1500 rpm commanded speed. Statistical uncertainty, possible dependence between records, and any unverified condition assignments remain separate from the physical plausibility of the proposed damage interpretation. Further validation on traceable, independently acquired tests, together with measurements of contact, deformation, and temperature, would be needed before treating the model as a general predictor of UHMWPE TSA durability.

## 5. Conclusions

This study investigated the cyclic durability of UHMWPE twisted string actuators and developed a second-order log-polynomial model for predicting lifetime as a function of string diameter, string number, and applied mass. The experimental results showed that increasing string diameter and string number generally improved cyclic lifetime, whereas increasing applied mass reduced durability. The second-order model achieved a lower average leave-one-condition-out cross-validation error than the reduced power-law model (2.8% versus 3.4% MAPE), although the improvement was not uniform across all conditions. From an engineering perspective, the proposed model provides a practical basis for screening string configurations according to a specified load and target lifetime. For example, at an applied mass of 20 kg and a string diameter of 1.5 mm, the model predicts approximately 32,000 cycles for two strings and 74,000 cycles for four strings. Thus, for a target lifetime of 50,000 cycles, the four-string configuration would be a more suitable candidate within the tested configurations. These predictions are intended for preliminary design comparison rather than guaranteed service-life estimates, and suitable safety margins and experimental verification remain necessary. The local sensitivity analysis indicated that lifetime depends on the combined effects of string geometry and applied loading. Post-failure observations also revealed filament damage and surface wear consistent with repeated contact and sliding. However, these observations do not establish a unique failure mechanism or independently quantify the contributions of friction and time-dependent deformation. The applicability of the proposed model is limited to the investigated LIROS D-Pro SK78 string configurations: diameters of 1.0, 1.5, and 2.0 mm; string-number ranges of 2–7, 2–5, and 2–4, respectively; and applied masses of 15, 20, and 25 kg. The experiments used a 250 mm effective free length, an initial target contraction of 50 mm, and a commanded motor speed of 1500 rpm. Predictions outside the tested configurations or under different operating conditions require further experimental validation. Within these limitations, the proposed approach can support the preliminary selection of string configurations for durability-oriented TSA design.

## Figures and Tables

**Table 1:** Nominal diameters and manufacturer-reported specifications of LIROS D-Pro SK78 braided strings.

Nominal Diameter, *d* (mm)	Listed Mass per Unit Length (g/m)	Linear Density (tex)	Manufacturer-Listed Breaking Load (N)	Sources
1.0	0.9	900	1950	[24,25]
1.5	1.3	1300	2300	[24,26]
2.0	1.8	1800	4100	[24,27]

**Table 2:** Test matrix for the durability evaluation of the twisted string actuator according to string diameter, number of strings, and applied mass.

String Diameter, (d) (mm)	String Count, (n)	Applied Mass, (M) (kg)	Conditions	Replicates per Condition	Total Specimens
1.0	2–7	15, 20, 25	18	10	180
1.5	2–5	15, 20, 25	12	10	120
2.0	2–4	15, 20, 25	9	10	90
Total	–	–	39	–	390

**Table 3:** Fitted coefficients of the full second-order log-polynomial model (conditional OLS analysis).

Coefficient	Regressor	Estimate	95% OLS Interval
*β*_0_	1	11.216	[11.187, 11.245]
*β_d_*	*x_d_*	2.527	[2.449, 2.605]
*β_n_*	*x_n_*	1.258	[1.196, 1.321]
*β_M_*	*x_M_*	−1.322	[−1.396, −1.248]
*β_dd_*	$x_{d}^{2}$	−0.099	[−0.339, 0.141]
*β_nn_*	$x_{n}^{2}$	0.053	[−0.045, 0.152]
*β_MM_*	$x_{M}^{2}$	−0.431	[−0.827, −0.035]
*β_dn_*	*x_d_x_n_*	0.0751	[−0.074, 0.224]
*β_dM_*	*x_d_x_M_*	0.359	[0.141, 0.576]
*β_nM_*	*x_n_x_M_*	−0.012	[−0.166, 0.142]

**Table 4:** Matched performance comparison of the reduced power-law and second-order models.

Metric	Reduced Power-Law (Matched Refit)	Second-Order (Matched Fit)
Number of regression coefficients	4	10
$R_{y}^{2}$ (log-scale in-sample fit)	0.997	0.998
Adjusted $R_{y}^{2}$ (log-scale fit)	0.997	0.998
RMSE (cycles; in-sample)	2432.270	1766.160
MAPE (%; in-sample)	3.076	2.121
$R_{N}^{2}$ (held-out cycle scale)	0.996	0.997
RMSE (cycles; condition-wise CV)	2748.150	2357.230
MAPE (%; condition-wise CV)	3.443	2.827
*R*^2^ (cycle-scale in-sample)	0.997	0.998
Maximum APE (%; condition-wise CV)	13.959	14.206
AICc (Gaussian log-residual model)	−125.838	−127.366
BIC (Gaussian log-residual model)	−119.338	−118.845

## Data Availability

The original contributions presented in this study are included in the article. Further inquiries can be directed to the corresponding author.
